# The role of SET/I2PP2A in canine mammary tumors

**DOI:** 10.1038/s41598-017-04291-7

**Published:** 2017-06-27

**Authors:** Satoru Kake, Shunya Tsuji, Shuhei Enjoji, Sayaka Hanasaki, Hiroshi Hayase, Ryotaro Yabe, Yuiko Tanaka, Takayuki Nakagawa, Hao-Ping Liu, Shih-Chieh Chang, Tatsuya Usui, Takashi Ohama, Koichi Sato

**Affiliations:** 1 Laboratory of Veterinary Pharmacology, Joint Faculty of Veterinary Medicine, Yamaguchi University, Yamaguchi, Japan; 2 Department of Comparative Animal Science, College of Life Science, Kurashiki University of Science and The Arts, Okayama, Japan; 3 The Laboratory of Veterinary Surgery and the Veterinary Medical Center, Graduate School of Agricultural and Life Sciences, The University of Tokyo, Tokyo, Japan; 4 Department of Veterinary Medicine, College of Veterinary Medicine, National Chung Hsing University, Taichung, Taiwan; 5 Department of Veterinary Medicine, Veterinary Medical Teaching Hospital, College of Veterinary Medicine, National Chung Hsing University, Taichung, Taiwan; 6 Laboratory of Veterinary Toxicology, Joint Faculty of Veterinary Medicine, Yamaguchi University, Yamaguchi, Japan

## Abstract

Canine mammary tumor is the most common neoplasm in female dogs, and it has generated considerable attention as a translational model for human breast cancer. Ser/Thr protein phosphatase 2A (PP2A) plays a critical role as a tumor suppressor, and SET/I2PP2A, the endogenous inhibitory protein of PP2A, binds directly to PP2A and suppresses its phosphatase activity. Here, we investigated the role of SET in the tumorigenic growth in canine mammary tumor as well as in the sensitivity of tumors to existing therapeutics. Elevated protein levels of SET were observed in advanced-stage of canine mammary tumor tissues of dogs compared with paired normal tissues. Knockdown of SET expression in a canine mammary tumor cell line CIP-m led to increased PP2A activity and decreased cell proliferation, colony formation, and *in vivo* tumor growth. We observed suppression of mTOR, β-catenin, and NFκB signaling by SET knockdown. The sensitivity of CIP-m cells to doxorubicin was decreased by SET knockdown, while SET knockdown in CIP-m cells did not affect sensitivity to 4-OH-tamoxifen, carboplatin, bortezomib, and X-ray radiation. These data suggest that SET plays important roles in the tumor progression of a subset of canine mammary tumor by suppressing PP2A activity and enhancing mTOR, β-catenin, and NFκB signaling.

## Introduction

Canine mammary tumor is one of the most common diseases in small animal medicine and accounts for ~40% of tumors in female dogs. Canine mammary tumor has also generated considerable attention as a translational model for human breast cancer^1^. Age, heredity, hormones, and obesity are reportedly risk factors of breast cancer^2, 3^, and surgical resection remains the first choice for mammary tumor therapy. However, for infiltrative and metastatic cases, adjuvant chemotherapy using agents such as doxorubicin and carboplatin; or radiation therapy are performed^4, 5^. Endocrine therapy using the estrogen receptor modulator tamoxifen is effective for a subset of human breast cancer, but treatment with tamoxifen in dogs with mammary tumors produces estrogen-like side effects^6^. For, Her2-positive human breast cancer, tyrosine kinase inhibitors have been shown to have certain therapeutic effect. There are currently no effective chemotherapy protocols that yield entirely satisfactory outcomes, especially in the case of so called triple-negative breast cancer; thus, novel targets for canine mammary tumor/ human breast cancer therapy must be investigated.

Protein phosphorylation is an essential regulatory mechanism for cell signaling and is reversibly regulated by protein kinases and protein phosphatases. Protein phosphatase 2A (PP2A) is an evolutionarily conserved serine/threonine phosphatase that regulates a wide range of biological processes including the cell cycle and apoptosis^7^. PP2A is a crucial tumor suppressor and suppresses the tumorigenic characteristics of cells including disordered cell proliferation and stemness, by suppressing various tumor-promoting signals such as c-Myc, Akt/PKB, ERK1/2, mTORC1/p70 S6 kinase (p70S6K), Wnt/β-catenin, and NFκB^8–10^. In many human tumors, PP2A activity is suppressed by increased protein levels of endogenous PP2A inhibitory proteins such as SET, CIP2A, and PME-1^11^.

The human *set* gene was discovered as a component of the *set-can* fusion gene, which was produced by a somatic translocation in acute undifferentiated leukemia^12^, and two human SET isoforms (α and β) with high homology have been identified^13^. We previously cloned four isoforms of canine SET (SETα, β, γ and δ) and showed that the canine SETα protein shares ~94% homology with human SETα^14^. Enhanced SET expression has been observed in many human tumors including breast cancer, chronic myeloid leukemia, and B-cell non-Hodgkin lymphoma, and SET expression levels have moreover been shown to correlate positively with poor prognosis of chronic myeloid leukemia^15, 16^. In human breast cancer, increased SET protein level has been reported to lead to c-Myc stabilization^16^. However, the role of SET in canine mammary tumor has not yet been studied. Here, we show that SET protein levels are elevated in canine breast cancer tissues, and that knockdown of SET expression in a canine mammary tumor cell line of metastatic origin leads to increase in PP2A activity and decreases in cell proliferation, colony formation, and anchorage-independent cell growth. We further analyzed the effects of SET KD on the sensitivity of tumors to existing therapeutics.

## Material and Methods

### Mammary Tumor Tissues: Ethics, consent, permissions

Tissues were obtained from client-owned 13 dogs with canine mammary tumor underwent surgical resection at the Veterinary Medical Center of the University of Tokyo and Veterinary Medicine Teaching Hospital of Collage of Veterinary Medicine, National Chung Hsing University, between January 2015 and November 2016. An Ethics Committee of the Veterinary Medical Center of the University of Tokyo and Veterinary Medicine Teaching Hospital of Collage of Veterinary Medicine, National Chung Hsing University granted ethical approval, all methods were performed in accordance with the relevant guidelines and regulations, and owners gave full informed consent. Breed, age, sex, and clinical stage of these cases are summarized in Table 1. The clinical stages of the patients were determined based on the WHO-TNM system of classification^17^. Approximately 5 mm cube tumor tissues were cut from the biggest mass, and normal mammary gland tissues were also collected from same individuals. These tissue samples were quickly snap-frozen and kept in −80 °C until examination.

**Table 1:** Characteristics of dogs with breast cancer

Dogs	Age	Sex	Breed	WHO Classification			
				T	N	M	Clinical Stage
1	10	Female	Chihuahua	1	0	0	I
2	7	Female	Welsh Corgi	3	0	0	III
3	11	Female	Miniature Dachshund	1	0	0	I
4	11	Female	Chihuahua	2	0	0	II
5	12	Female	Papillon	1	0	0	I
6	12	Female	Maltese	1	0	0	I
7	10	Female	Miniature Dachshund	1	0	0	I
8	5	Female	Japanese Chin	1	0	0	I
9	13	Female	Welsh Corgi	3	1	1	V
10	16	Female	Miniature Dachshund	3	0	1	V
11	8	Female	Mixed	1	0	0	I
12	8	Female	Shiba	3	0	0	III
13	15	Female	Afghan	1	1	0	IV

### Cell Culture

Canine mammary tumor cell lines CIP-p (primary origin) and CIP-m (metastatic origin), which previously generated from the same individual (Shih Tzu)^18^, were cultured in RPMI 1640 containing 10% FBS and 1x antibiotic/antimycotic (Life Technologies, Carlsbad, CA, USA).

### Antibodies

Antibodies were obtained from the indicated supplier: anti-phospho-Tyr307 PP2Ac (ab32104) (Abcam, MA, USA), anti-total PP2Ac (07-324) (Merck Millipore, MA, USA), p97/VCP (GTX113030) (GeneTex, CA, USA), anti-tubulin alpha (RB-9281-P0) (Thermo Scientific, MA, USA), anti-β-catenin (610153), anti-E-cadherin (610181) (BD Biosciences, San Jose, CA, USA), anti-SET (sc-5655), anti-total GSK3β (sc-9166), anti-total p65 NFκB (sc-372), anti-total p70S6K (sc-230) (Santa Cruz Biotech, Santa Cruz, CA), anti-total ERK p42/p44 (9107), anti-phospho ERK p42/p44 (9101), anti-total Akt (2920), anti-phospho-Ser473 Akt (4060), anti-c-Myc (5605), anti-phospho-Ser9 GSK3β (5558), anti-IκBα (4814), anti-phospho-Ser536 p65 NFκB (3033), anti-N-cadherin (13116), and anti-phospho-Thr389 p70S6K (9234) (Cell Signaling, MA, USA).

### shRNA and Lentivirus Production

shRNA sequences and procedure for lentivirus production was previously described^19^. Briefly, to produce lentiviruses, 3 *μ*g of pLVSIN, 2.3 *μ*g of a packaging plasmid (psPAX2) and 1.3 *μ*g of a coat-protein plasmid expressing vesicular stomatitis virus G protein (pMD2.G) were transfected into Lenti-X 293T cells cultured in 60-mm dishes using PEI Max (Polysciences, PA, USA) according to the manufacturer’s instruction. Viral supernatants were collected after 48 hr, and after filtering (0.22 *μ*m), were added to cells for 8 hr.

### Immunoblotting

Immunoblotting was performed as previously described^20^. Briefly, cells were lysed in a buffer containing 50 mM Tris-HCl (pH 8.0), 5 mM EDTA, 5 mM EGTA, 1% Triton X100, 1 mM Na_3_VO_4_, 20 mM sodium pyrophosphate and Roche’s complete protease inhibitor cocktail. The proteins were separated by SDS-PAGE and transferred onto nitrocellulose membrane (Wako, Osaka, Japan). Membranes were blocked with 0.5% skim milk and treated with primary antibodies. Immunoreactive bands were detected using Western Lightning ECL Pro (PerkinElmer, Freiburg, Germany) and visualized using a LAS-3000 luminescent image analyzer (Fujifilm, Tokyo, Japan).

### Cell Proliferation and Cell Viability Assay

1.0 × 10^3^ cells were seeded on 24-well plates and cultured for 4 days. Cell Counting Kit-8 (CCK8, Dojindo, Kumamoto, Japan) was used to analyze cell proliferation according to the manufacturer’s instruction. For cell viability analysis, 1.0 × 10^3^ cells were seeded on 24-well plate, and drugs were added to the medium after 24 hr. After additional 48 hr, CCK8 was used to analyze cell viability.

### Measurement of Doubling Time

1.0 × 10^4^ cells were seeded on 4 chamber 35-mm glass bottom dishes. After 24 hr, phase difference images were taken every 15 min by BZ-9000 fluorescence microscopy (Keyence, Osaka, Japan) at 37 °C in a 5% CO_2_ atmosphere for 48 hr, and doubling time of cells was analyzed.

### Colony Formation Assay

3.0 × 10^2^ cells were seeded on 60-mm dishes. After 1 week, cells were fixed with 99.5% ethanol, colonies were stained with Giemsa solution, and the number of colonies was counted.

### Soft Agar Colony Formation Assay

Soft agar colony formation assay was performed for the analysis of anchorage-independent cell growth. A 6-well plate was covered with 2.5 ml bottom agar (RPMI-1640 containing 10% FBS, 2.8% NaHCO_3_, 1x antibiotic/antimycotic and 0.75% agarose) and solidified by cooling at 4 °C. 6 × 10^3^ cells with 1.5 ml top agar (RPMI-1640 containing 10% FBS, 2.8% NaHCO_3_, 1x antibiotic/antimycotic and 0.36% agarose) was added. After 3 weeks of culture, crystal violet was used to stain the cells and number of colonies was counted.

### Xenograft of Canine Mammary Tumor Cell Line

A total of 3 × 10^6^ cells of CIP-m cells were xenografted into the hind legs of NOD.CB17 *Prkdc* ^*scid*^/J mice. Tumors were measured by a caliper and tumor volume was calculated by ((width + length)/4)^3^ × 3 × 4/3.

### PP2A Activity Assay

PP2A activity assay was performed using Active PP2A DuoSet IC kit (R&D systems, MN, USA) according to the manufacturer’s instruction.

### X-ray Irradiation Assay

For X-ray irradiation, MX-80 Labo desktop X-ray irradiator (mediXtec, Chiba, Japan) was used. 1 × 10^4^ cells were seeded on a 24-well plate and X-ray irradiation (2 Gy or 4 Gy) was applied on the cells. 3.0 × 10^2^ cells were seeded on 60-mm dishes and cultured for 1 week. Cells were fixed with 99.5% ethanol, colonies were stained with Giemsa solution, and the number of colonies was counted.

### Statistical Analysis

Statistical analysis was performed using SigmPlot (HULINKS). The results are expressed as means ± S.E. Student’s t test was used for comparison between two groups. Groups more than three were compared using one-way analysis of variance, after which Fisher LSD test was used. For all analyses, a probability value of p < 0.05 was considered statistically significant.

## Results

### SET protein levels are enhanced in advanced-stage of canine mammary tumor tissues and cell lines

**Figure 1:**
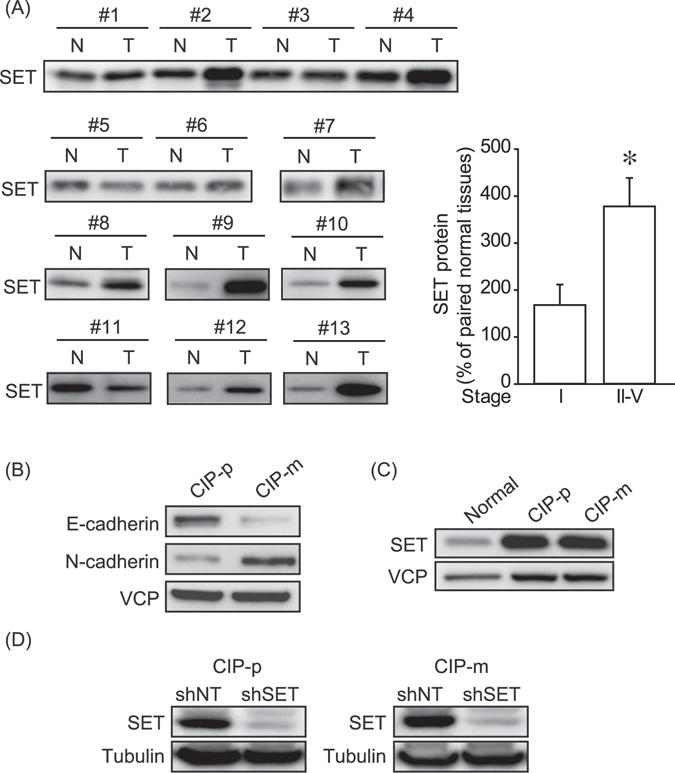
SET protein levels in canine mammary tumor tissues and cell lines. (**A**) 10 μg of proteins of canine mammary tumor tissues and paired normal mammary gland tissues were subjected to analyze SET protein levels by immunoblotting. Band densities of cancer tissues were normalized to paired normal tissues as 100%. (**B**) EMT markers protein levels of canine mammary tumor cell lines were determined by immunoblotting. (**C**) SET protein levels of canine mammary tumor cell lines were determined by immunoblotting. Normal: normal mammary gland tissue. p97/VCP was used as a loading control. (**D**) The effects of non-targeting shRNA (shNT) and SET targeting shRNA (shSET) were determined by immunoblotting. Representative pictures from 2 independent experiments were shown. Tubulin was used as a loading control.

Although SET is frequently overexpressed in human breast cancer, it was previously unknown whether SET protein levels are enhanced in canine mammary tumor tissues. Thirteen mammary tumor tissues and paired normal tissues from the same dogs brought to the Veterinary Medical Center, The University of Tokyo, and Veterinary Medicine Teaching Hospital of Collage of Veterinary Medicine, National Chung Hsing University were collected (Table 1), and from each of these tissue samples 10 *μ*g proteins was subjected to analyze the SET protein level by immunoblotting. SET protein levels in tumor tissues were normalized with paired normal tissues as 100%. SET protein levels were found to be significantly elevated in stage II-V mammary tumor tissues compared with stage I mammary tumor tissues (Fig. 1A).

Next, the SET protein levels in canine mammary tumor cell lines, CIP-p and CIP-m, were examined. CIP-p and CIP-m are of primary and metastatic origin, respectively, from the same individual. As shown in Fig. 1B, we observed that CIP-p and CIP-m cells have epithelial phenotype and mesenchymal phenotype, respectively, which was shown by the protein levels of E-cadherin (epithelial marker) and N-cadherin (mesenchymal marker). Both cell lines showed higher SET protein levels compared with normal mammary gland tissues (Fig. 1C). The canine mammary tumor cells were infected with lentivirus to stably express non-target shRNA (shNT) and SET targeting shRNA (shSET). We obtained polyclonal populations of these cell lines, and as shown in Fig. 1D, shSET effectively suppressed SET protein expression in both CIP-p and CIP-m cells. SET expressions were stably inhibited at least during day 7–30 following lentivirus infection.

### SET knockdown activates PP2A and suppresses the tumorigenic growth of canine mammary tumor cells

**Figure 2:**
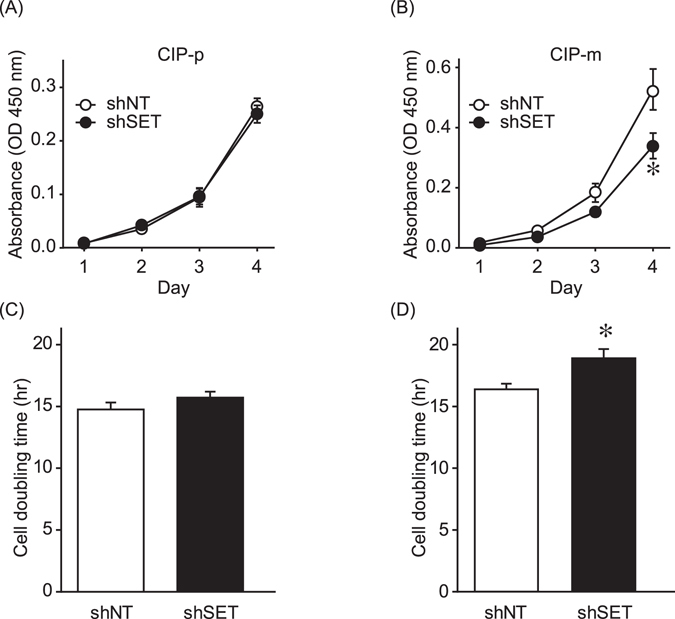
SET knockdown suppressed proliferation of canine mammary tumor cells. (**A** and **B**) Cell proliferation of CIP-p (**A**) and CIP-m (**B**) expressing shNT or shSET was determined by Cell Counting Kit-8. Quantitative data from 3 independent experiments performed duplicate are shown. *P < 0.05 vs. shNT. (**C** and **D**) Cell doubling time of CIP-p (**A**) and CIP-m (**B**) expressing shNT or shSET was determined as described in Material and Methods. 15–17 cells from 2 independent experiments were analyzed. *P < 0.05 vs. shNT.

In human breast cancer cell lines, SET knockdown (KD) suppressed cell proliferation and anchorage-independent cell growth^16^. Therefore, the effects of SET KD on the tumorigenic growth of canine mammary tumor cell lines were assessed. The cell proliferation of CIP-m cells (metastatic origin), but not of CIP-p cells (primary origin), was found to be inhibited by SET KD (Fig. 2A and B). We also observed that doubling time of CIP-m cells were delayed by SET KD, but SET KD did not affect the doubling time of CIP-p cells (Fig. 2C and D).

**Figure 3:**
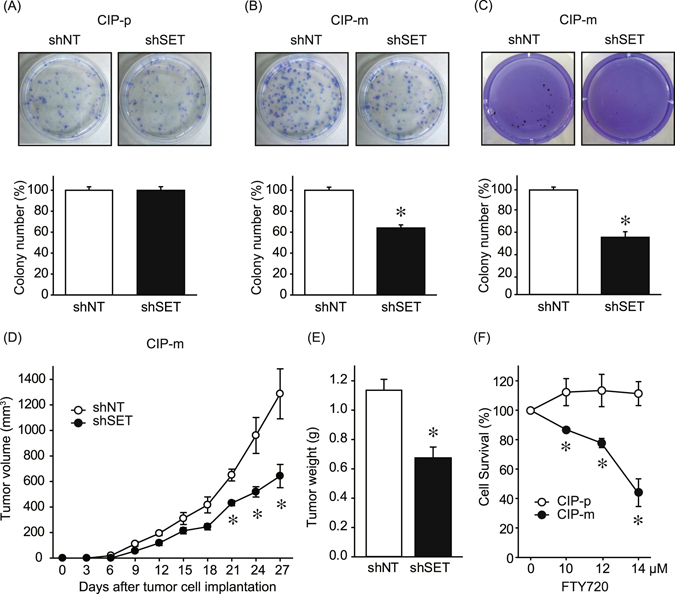
SET knockdown suppressed colony formation ability and anchorage-independent cell growth of canine mammary tumor cells. (**A** and **B**) Colony formation ability was examined for CIP-p (**A**) and CIP-m (**B**) expressing shNT or shSET. Colony numbers of shSET expressing cells were normalized to colony numbers of shNT expressing cells as 100%. Quantitative data from 3 independent experiments performed duplicate are shown. *P < 0.05 vs. shNT. (**C**) Soft agar colony formation assay was performed to examine the anchorage-independent growth of CIP-m cells expressing shNT or shSET. Colony numbers of shSET expressing cells were normalized to colony numbers of shNT expressing cells as 100%. Quantitative data from 3 independent experiments performed duplicate are shown. *P < 0.05 vs. shNT. (**D** and **E**) *In vivo* tumor growth of CIP-m expressing shNT or shSET was examined in a xenograft model. Tumor growth was monitored by measuring tumor volume (**D**). Tumors were harvested after 35 days growth, and tumor weights were measured (**E**). N = 7. *P < 0.05 vs. shNT. (**F**) Cell viability of CIP-p and CIP-m cells were analyzed by CCK8 after 48 hr treatment with FTY720. Cell viabilities were normalized to non-treated control of each cell lines as 100%. Quantitative data from 2 independent experiments performed duplicate are shown.

Consistent with cell proliferation data, we observed SET KD decreased CIP-m colony numbers, but not CIP-p colony numbers, in a colony formation assay (Fig. 3A and B). SET KD was shown to suppress anchorage-independent cell growth of CIP-m cells (Fig. 3C). CIP-p cells formed very small number of colonies even in shNT expressing cells. Furthermore, we found that SET KD inhibited *in vivo* tumor growth of CIP-m cells in the xenograft model (Fig. 3D and E). FTY720 (Fingolimod) is a sphingosine analog used as an immunosuppressant in human multiple sclerosis patients, and has been reported to directly interact with SET and recover PP2A activity^21^. FTY720 dose dependently killed CIP-m cells, but did not affect CIP-p cell survival (Fig. 3F). These data suggest that the role of SET in cells changes during cancer progression and that SET plays a tumor-promoting role in a subgroup of canine mammary tumor cells.

**Figure 4:**
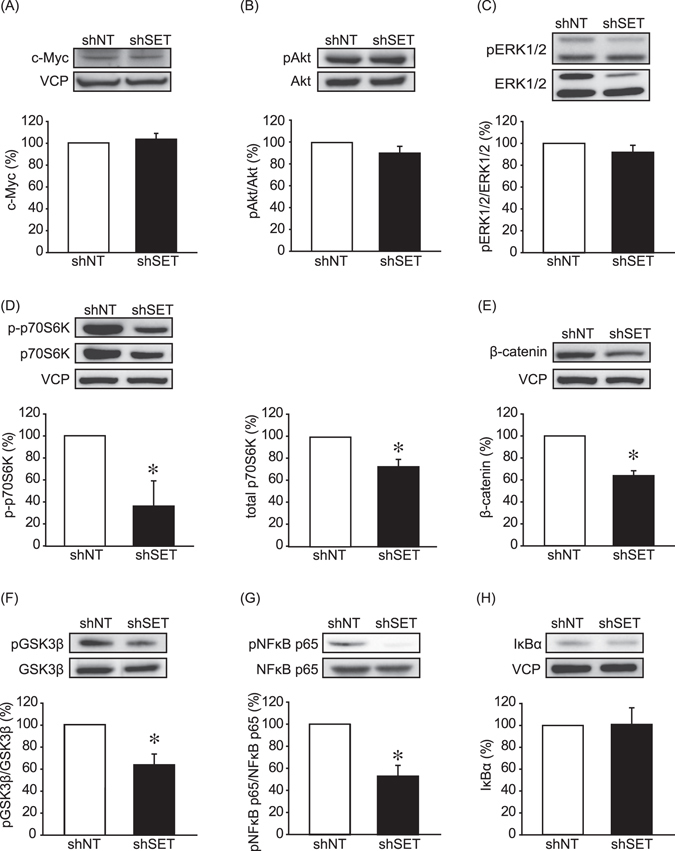
SET knockdown suppressed mTORC1/p70S6K, β-catenin, and NFκB signaling. Levels of phosphorylation/protein of c-Myc (**A**), ERK1/2 (**B**), Akt (**C**), p70S6K (**D**), β-catenin (**E**), GSK3β (**F**), NFκB p65 (**G**), and IκBα (**H**) of CIP-m cells were determined by immunoblotting. Quantitative data from 3–6 independent experiments are shown. *P < 0.05 vs. shNT.

Immunoblotting was performed to investigate the effect of SET KD on cell signaling (Fig. 4). Because SET KD has been reported to decrease the protein levels of c-Myc and the phosphorylation levels of Akt in human breast cancer cells^16, 22^, we first examined the effects of SET KD on these signaling molecules in CIP-m cells (Fig. 4A,B). No differences were detected in the c-Myc protein and Akt phosphorylation and protein levels between shNT and shSET expressing CIP-m cells, and SET KD moreover did not decrease ERK1/2 phosphorylation and protein levels (Fig. 4C), suggesting that SET does not affect these signaling pathways in CIP-m cells. We previously reported that SET KD suppresses p70 S6 kinase (p70S6K) phosphorylation, a marker for mTORC1 (mammalian target of rapamycin complex 1) signaling activation, in canine melanoma cells^19^. Consistent with our previous finding, SET KD in the present study resulted in decreased phosphorylated p70S6K in canine mammary tumor cells (Fig. 4D). To note, total protein level of p70S6K was slightly but significantly suppressed. Phospho/total p70S6K ratio was also decreased by SET KD (68.57 ± 2.81%, *P* < 0.05 vs. shNT).

In human breast cancer cells, Wnt/β-catenin and NFκB signaling is frequently found to be upregulated^23, 24^. The activation of Wnt/β-catenin signaling is characterized by the stabilization of cytosolic β-catenin by decreased phosphorylation of β-catenin. Activation of PP2A has been reported to activate GSK3β by Ser9 dephosphorylation that in turn leads to β-catenin phosphorylation/degradation^25^. In this study, we observed decreased β-catenin protein levels and GSK3β phosphorylation levels by SET KD (Fig. 4E and F). SET KD did not affect total protein expression of GSK3β. We also observed decreased protein levels of cyclin D1, one of the target of β-catenin signaling (Fig. S1). PP2A is known to negatively regulate NFκB activity via the dephosphorylation of Ser536 of NFκB p65^10^. Here we show that SET KD decreased the phosphorylation levels of NFκB p65 without affecting the NFκB p65 and IκBα protein levels (Fig. 4G and H). The effects of SET KD on cell signaling in CIP-p cells (Fig. S2) were also investigated, and it was found that SET KD had little effect in CIP-p cells: although p70 S6K phosphorylation was significantly decreased, the level of inhibition was lower than that in CIP-m cells. Interestingly, IκBα protein levels were increased by SET KD in CIP-p cells.

**Figure 5:**
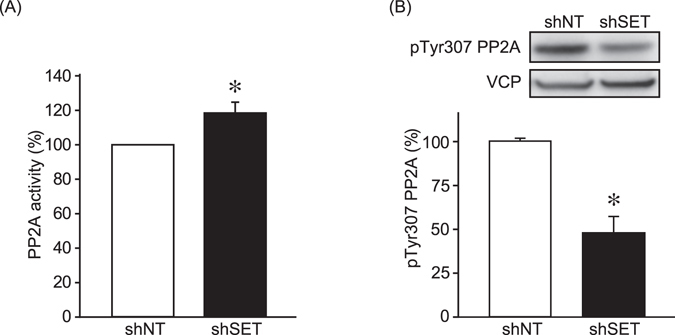
SET knockdown enhanced PP2A activity. (**A**) PP2A phosphatase activity of CIP-m cells was analyzed as indicated in material and methods. Quantitative data from 3 independent experiments performed duplicate are shown. *P < 0.05 vs. shNT. (**B**) PP2A Tyr307 phosphorylation levels of CIP-m cells were determined by immunoblotting. Band density of phospho-Try307 was normalized with the band density of shNT expressing CIP-m cells as 100%. VCP was used as a loading control. Quantitative data from 4 independent experiments are shown. *P < 0.05.

Because SET is the inhibitory protein for PP2A, the effects of shSET on PP2A activity were investigated. Enhanced PP2A activity was observed in CIP-m cells expressing shSET (Fig. 5A). Tyr307 phosphorylation of PP2A is known to suppress PP2A activity^26^. Consistent with the PP2A activity data shown here, shSET significantly decreased the levels of Tyr307 phosphorylation (Fig. 5B). These data suggest that SET enhanced mTORC1/p70S6K, β-catenin and NFκB signaling via the suppression of PP2A activity.

### Effects of SET KD on mammary tumor therapy

**Figure 6:**
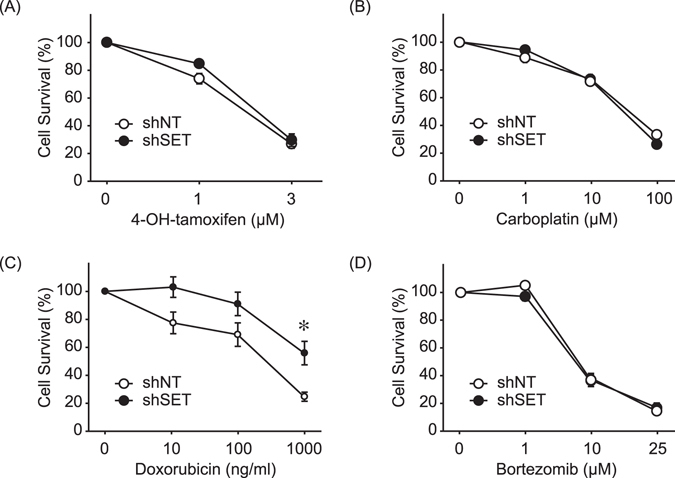
SET knockdown renders the canine breast cancer cells resistant to doxorubicin. Cell viability of CIP-m expressing shNT or shSET was analyzed by CCK8 after 48 hr treatment with 4-OH-tamoxifen (**A**), carboplatin (**B**), doxorubicin (**C**), and bortezomib (**D**). Cell viabilities were normalized to non-treated control of each cell lines as 100%. Quantitative data from 3 independent experiments performed duplicate are shown. *P < 0.05 vs. shNT.

SET KD has been reported to sensitize A549 human lung cancer cells to cisplatin^27^, suggesting a role of SET in the anti-tumor effects of chemotherapies. We therefore investigated the effects of SET KD on existing breast cancer therapies. Cells were treated with the estrogen receptor modulator 4-OH-tamoxifen (1 and 3 μM), carboplatin (1, 10, and 100 μM), doxorubicin (10, 100, and 1000 ng/ml), or bortezomib (1, 10, and 25 μM) for 48 hr (Fig. 6). Bortezomib, a potent inhibitor of the 26S proteasome with broad anti-tumor effects, reportedly decreases the expression of CIP2A, another PP2A inhibitory protein, to exert anti-tumor effects on human triple negative breast cancer cells^28^. In CIP-m cells, however, SET KD did not alter sensitivities to 4-OH-tamoxifen, carboplatin, or bortezomib. Contrary to our prediction, SET KD rendered cells resistant to doxorubicin, suggesting that the anti-tumor effects of doxorubicin are, to some extent, dependent on SET.

**Figure 7:**
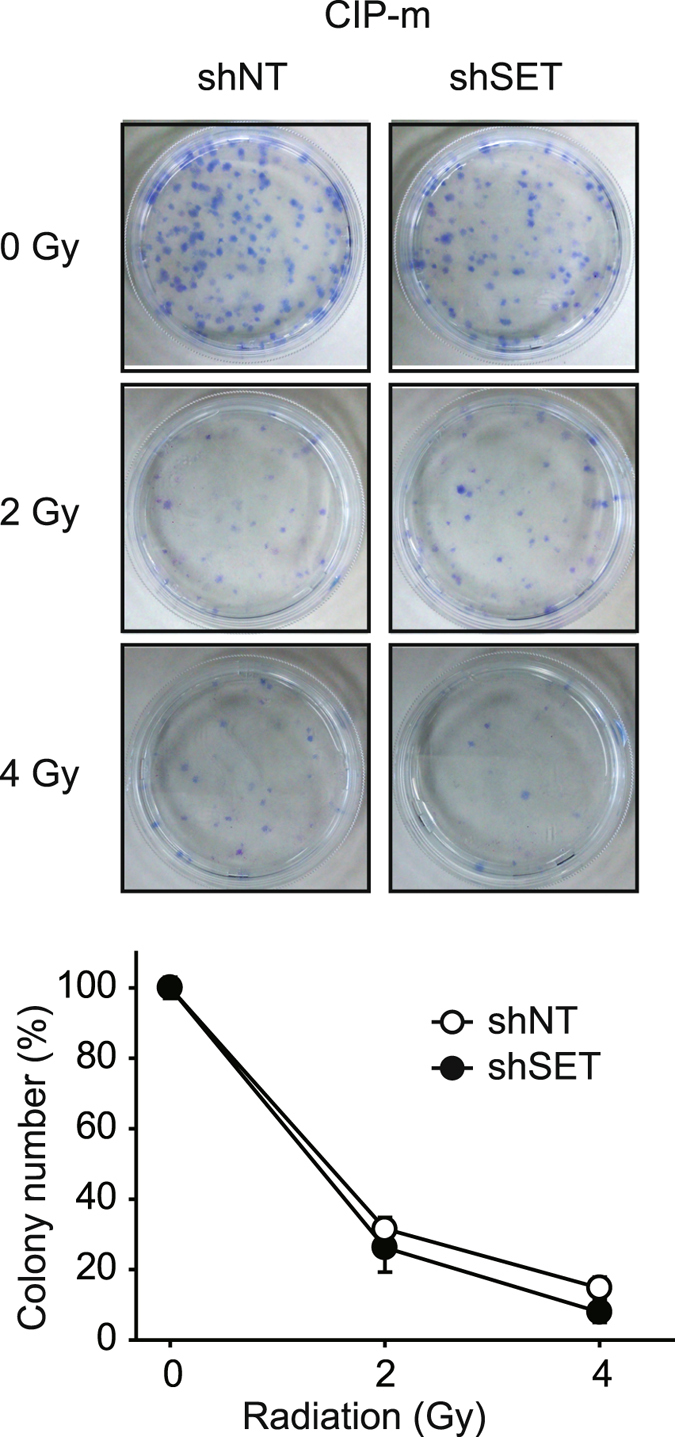
SET knockdown does not affect the sensitivity for radiation therapy. Colony formation ability of CIP-m cells expressing shNT or shSET after exposure to X-ray radiation (2 and 4 Gy) was analyzed. Representative pictures and quantitative data normalized with 0 Gy as 100% were shown. Quantitative data from 3 independent experiments performed duplicate are shown.

The effects of SET KD on radiation therapy were also investigated in this study (Fig. 7), specifically by assessing the colony formation ability of cells exposed to X-ray radiation. As expected, radiation treatment decreased the colony formation ability of CIP-m cells; however, no significant differences in colony formation were observed between shNT and shSET cells, suggesting that SET does not play a role in the cells’ resistance to radiation therapy.

## Discussion

The aim of this study was to investigate the role of SET in the tumorigenic characteristics and the effects of existing therapeutics on canine mammary tumor, which is thought to be a good translational model for human breast cancer. In this study, SET protein levels were found to be elevated in advanced-stage of canine mammary tumor tissues. Knockdown on SET expression was shown to decrease the tumorigenic growth of metastasis-derived CIP-m cells, but not that of primary origin CIP-p cells. Consistent with our data, SET KD is known to suppress the proliferation of human breast cancer cells^16^. The molecular mechanisms driving these decreases in proliferation, however, differ between previous reports and our findings. In human breast cancer cells, SET KD has been shown to decrease c-Myc protein levels by enhancing the dephosphorylation of c-Myc Ser62^16^, while in this study SET KD did not alter c-Myc protein levels in canine mammary tumor cells. Similarly, SET KD suppressed ERK1/2 and Akt activity in human non-small cell lung cancer cells^29^; however, ERK1/2 and Akt phosphorylation levels in canine mammary tumor cells were not affected by SET KD in the present study. The reason for these differences remain unknown; however differences between species may play a role, such as the fact that dogs specifically express some carboxy terminal-truncated SET isoforms^14^. In this study, SET KD was found to enhance PP2A activity and suppress mTORC1/p70S6K, β-catenin, and NFκB signaling. It has been reported that PP2A associates directly with p70S6K and that KD of one of the regulatory subunits of PP2A enhances p70S6K Thr389 phosphorylation^9, 30^. The protein level of β-catenin is regulated by its phosphorylation by GSK3β, which is known to dephosphorylate/activate PP2A^25^. PP2A negatively regulates NFκB activity by dephosphorylating Ser536 of NFκB p65^10^. The protein level of the NFκB inhibitory protein IκBα was not affected by SET KD in this study, suggesting direct dephosphorylation of NFκB p65. Although further investigation is required to confirm this, our findings suggest that PP2A activation by SET KD suppressed mTORC1/p70S6K, β-catenin, and NFκB signaling in the canine mammary tumor cells. To our knowledge, this is the first report to suggest that increased SET levels positively regulate β-catenin and NFκB signaling in cancer cells. It is possible that these signaling pathways are also upregulated in human cancer cells with high SET expression, including breast cancer cells.

CIP-p (primary origin) and CIP-m (metastatic origin) were previously generated from the same individual (Shih Tzu)^18^. CIP-p cells form small number of colonies in soft agar colony formation assay (data not shown), suggesting that CIP-p cells is likely to be a lower grade cancer than CIP-m. Even though SET protein levels do not differ markedly between CIP-p and CIP-m cells, the effects of SET KD are different between these cell lines. The molecular mechanism governing the difference for the effects of SET KD is unclear; however, additional genetic mutations in CIP-m cells or differences in post-translational modifications of SET may be contributing factors.

In human breast cancer cells, the SET-targeting drugs OP449 and FTY720 (Fingolimod) reportedly to exert anti-tumor effects^16, 31^. We found that FTY720 selectively killed CIP-m cells in this work. We also reported that OP449 and FTY720 recovers PP2A activity and induces apoptosis in canine lymphoma and melanoma cells^19, 32^. Previously, SET KD was shown to sensitize A549 human non-small cell lung cancer cells to cisplatin by PP2A-dependent activation of the transcriptional factor NDRG1^27^. Moreover, FTY720 reportedly enhances the oxaliplatin–induced anti-tumor effects in human colon cancer cells^33^. However, in this study, SET KD did not sensitize CIP-m cells to carboplatin, another platinum-containing anti-tumor drug. FTY720 also reportedly enhances doxorubicin–induced anti-tumor effects in human colon cancer and breast cancer cells^31, 33^, while SET KD in the present study rendered cells resistant to doxorubicin. Doxorubicin synergistically exerts anti-tumor effect with histone deacetylase (HDAC) inhibitors, demonstrating the importance of chromatin remodeling in the effects of doxorubicin^34^. SET is reportedly involved in the regulation of chromatin remodeling and histone acetylation via a PP2A-independent mechanism^35, 36^. Because the anti-tumor effects of FTY720 depend on PP2A activation, the PP2A-independent function of SET may be responsible for this contradiction.

In the present study, the PP2A inhibitory protein SET/I2PP2A was shown to be highly upregulated in canine mammary tumor tissues. SET KD was furthermore shown to enhance PP2A activity; to inhibit mTORC1/p70S6K, β-catenin, and NFκB signaling; and to suppress the tumorigenic characteristics of a canine mammary tumor cell line. SET KD did not affect the sensitivity of cells to 4-OH-tamoxifen, carboplatin, bortezomib, and X-ray radiation, but did render cells resistant to doxorubicin. These findings support further investigation into the therapeutic potential of SET as a target for anti-tumor drugs for canine mammary tumor/human breast cancer.

## Electronic supplementary material

Supplementary Information
