# Supplementary material for: The role of SET/I2PP2A in canine mammary tumors
Source: Sci Rep. 2017 Jun 27;7:4279. doi: 10.1038/s41598-017-04291-7 (PMC5487328; doi:10.1038/s41598-017-04291-7)
Supplement: Supplementary file 1 — Supplementary Information [file 41598_2017_4291_MOESM1_ESM.pdf]

Supplementary Information

The role of SET/I2PP2A in canine mammary tumors

Satoru Kake[1, 2¶], Shunya Tsuji[1¶], Shuhei Enjoji[1], Sayaka Hanasaki[1], Hiroshi Hayase[1], Ryotaro Yabe[1], Yuiko Tanaka[3], Takayuki Nakagawa[3], Hao-Ping Liu[4], Shih-Chieh Chang[5], Tatsuya Usui[6], Takashi Ohama[1]*, Koichi Sato[1]

*Corresponding Author: t.ohama@yamaguchi-u.ac.jp (TO)
¶These authors contributed equally to this work

Supplement Figure 1

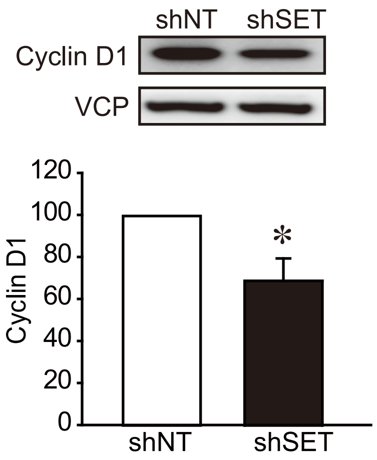

Figure S1 Effects of SET KD on cyclin D1 protein levels in CIP-m cells.

Cyclin D1 protein levels were determined by immunoblotting. Quantitative data from 3 independent experiments are shown. *: P<0.05 vs. shNT.

Supplement Figure 2

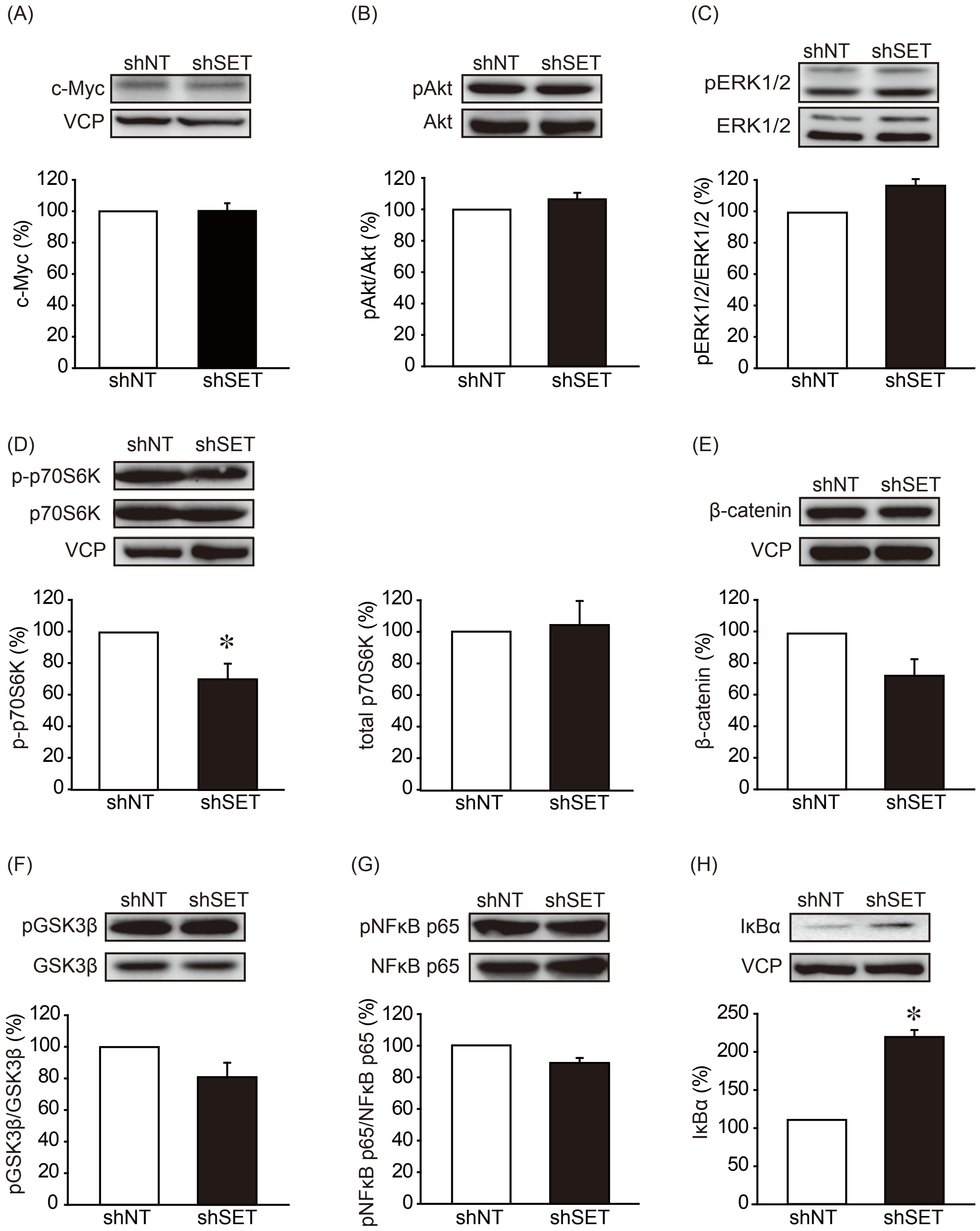

Figure S2 Effects of SET KD on cell signaling in CIP-p cells.

Levels of phosphorylation/protein of c-Myc (A), ERK1/2 (B), Akt (C), p70S6K (D), β-catenin (E), GSK3β (F), NFκB p65 (G), and IκBα (H) of CIP-p cells were determined by immunoblotting. Quantitative data from 3-6 independent experiments are shown. *: $P<0.05$ vs. shNT.

Supplement Figure 3

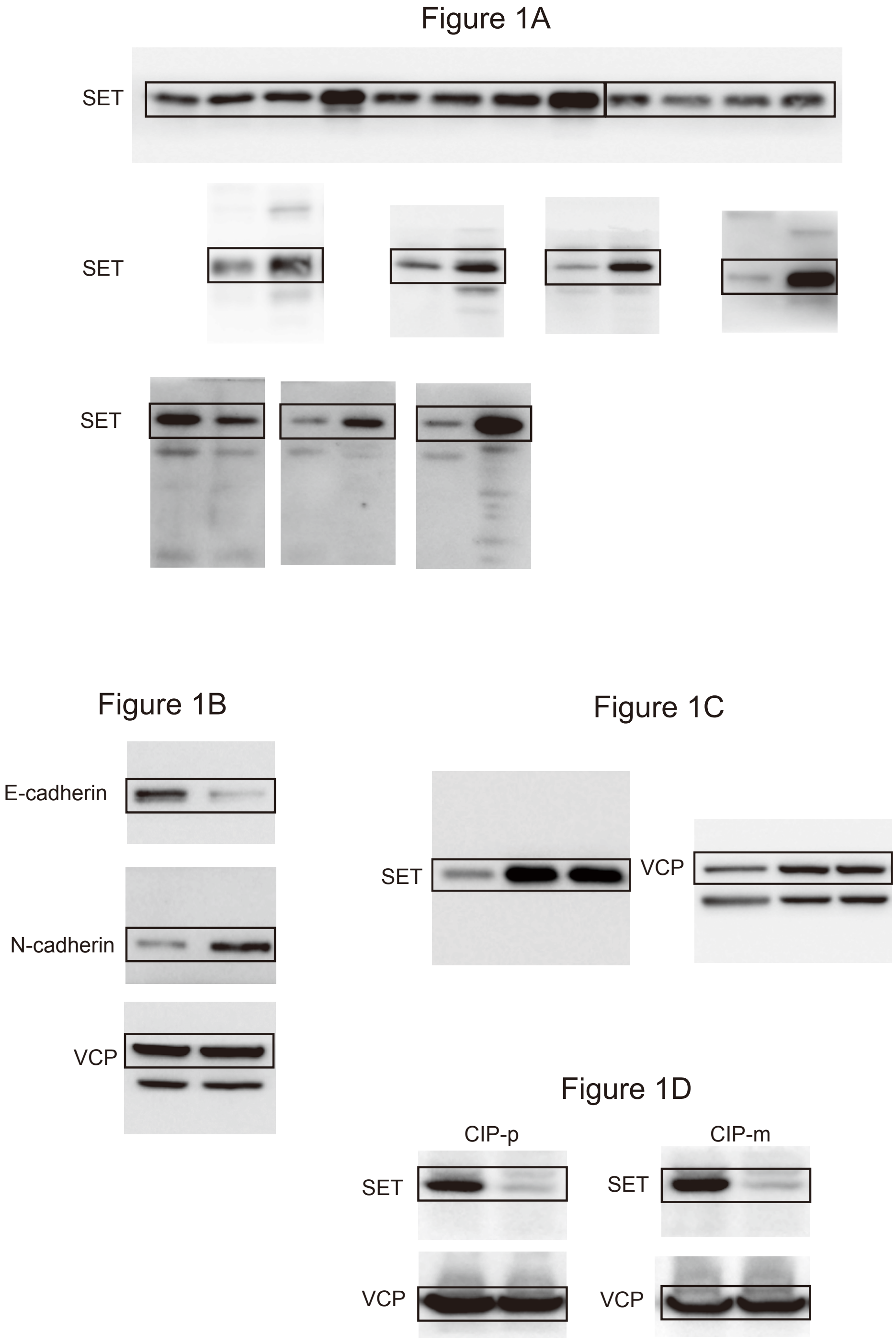

Figure S3 Uncropped images of Figure 1.

Supplement Figure 4

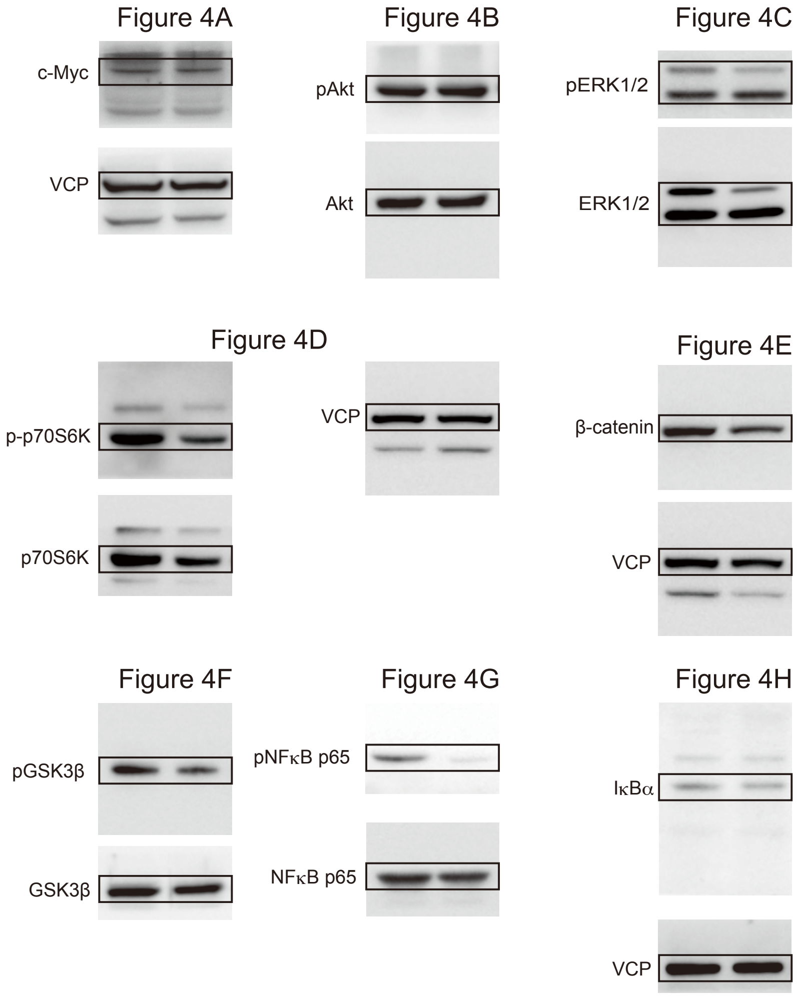

Figure S4 Uncropped images of Figure 3.

Supplement Figure 5

Figure 5B

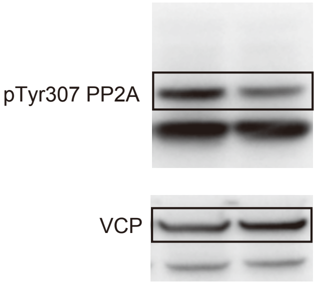

Figure S5 Uncropped images of Figure 5.
